# Development of 10-Hydroxycamptothecin-crizotinib conjugate based on the synergistic effect on lung cancer cells

**DOI:** 10.1080/14756366.2022.2132487

**Published:** 2022-10-28

**Authors:** Zhen Liu, Ye Xu, Lvqian Guo, Xinran Li, Junling Gao, Weiran Xie, Lianbo Zhao, Yuou Teng, Xuejiao Li, Peng Yu

**Affiliations:** a China International Science and Technology Cooperation Base of Food Nutrition/Safety and Medicinal Chemistry, Key Laboratory of Industrial Fermentation Microbiology of Ministry of Education, Tianjin Key Laboratory of Industry Microbiology, College of Biotechnology, Tianjin University of Science & Technology, Tianjin, P. R. China; b Central Laboratory, Endocrine and Metabolic Disease Center, The First Affiliated Hospital and College of Clinical Medicine of Henan University of Science and Technology, Medical Key Laboratory of Hereditary Rare Diseases of Henan; Luoyang Sub-Center of National Clinical Research Center for Metabolic Diseases, Luoyang, P. R. China

**Keywords:** 10-Hydroxycamptothecin, crizotinib, EGFR, NSCLC, conjugate

## Abstract

The effect of the combination of 10-Hydroxycamptothecin (HCPT) and crizotinib (CRI) on EGFR- and KRAS-mutant lung cancer cells was investigated and the conjugates of the two drugs were synthesised. HCPT combined with CRI synergistically inhibited the cell growth and proliferation of H1975, HCC827, and H460 without aggravating adverse effect on the normal cells. The combination synergistically enhanced the cell apoptosis rate through releasing Cyto-C by activation of Bcl-2 family-mediated mitochondrial signalling, which was associate with inactivating of EGFR related downstream signalling pathways including AKT, ERK, JNK, and p38 MAPK. Based on this synergy, the conjugates of HCPT and CRI (compounds **CH-1** and **CH-2**) with different chemical bonds were synthesised. Compound **CH-1** exhibited stronger cytotoxicity than HCPT and CRI alone or in combination. The combination of HCPT and CRI might be a promising therapeutic regimen and the conjugate **CH-1**was a potential target drug for the treatment of lung cancer.

## Graphical abstract

**Figure:**
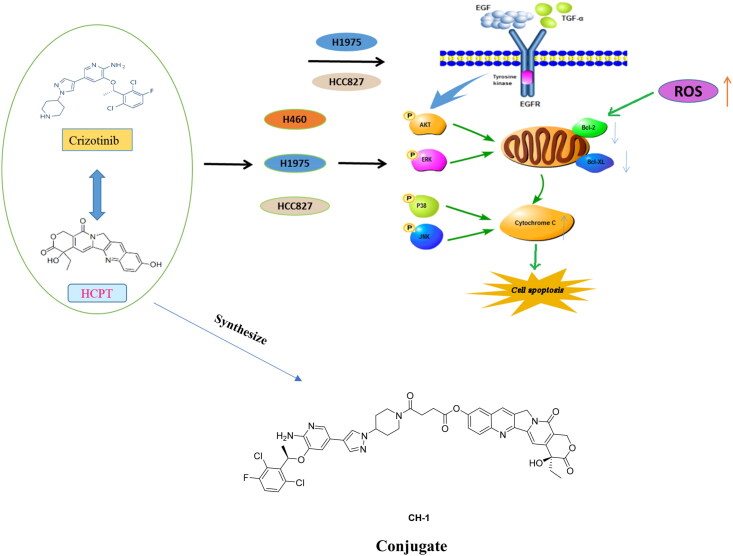

## 1. Introduction

Lung cancer is the most common malignancy and it is the leading cause of cancer-related deaths worldwide in 2020^1^, among which non-small cell lung cancer (NSCLC) accounts for 80–90%^2^. It was estimated that approximately 20–30% of lung cancer patients present with early-stage with the potential for curative-intent surgical resection and adjuvant platinum-based chemotherapy is a standard regimen but with a disappointing overall 5-year survival outcomes^3^. Various driver gene mutations of NSCLC were identified nearly two decades such as epidermal growth factor receptor (EGFR), anaplastic lymphoma kinase (ALK), V-Ki-ras2 Kirsten rat sarcoma viral oncogene (KRAS), C-ros oncogene 1 (ROS1), cellular mesenchymal-to-epithelial transition (c-Met), and so on^4^. Subsequently, drugs targeting driving mutations appeared including tyrosine kinase inhibitors (TKIs)^5^ and immune checkpoint inhibitors (ICIs)^6^. The targeted drugs especially EGFR tyrosine kinase inhibitors (EGFR-TKIs) bring significant benefits for the NSCLC patients harboured the mutant gene and improves the 5-year survival of patients. Unfortunately, all patients eventually develop acquired resistance to the first line EGFR-TKI and over 41% of them receive subsequent second line treatment, which also induce resistance and then the patients may receive third-line treatment, but there are only three generations of EGFR-TKIs at present^7^. Similarly, lung cancer patients often relapse due to drug resistance during the first 2 year of using other TKIs, such as ALK inhibitor crizotinib (CRI)^8^. Actually, the proportion of lung cancer patients with the driver mutations is relatively small, for instance, EGFR mutations account for 10–15% of NSCLC^7^, ALK rearrangements account for 2–5%, and ROS1 fusions occur in 1–4%^2^. That is, half of all NSCLC cases lack mutation detection. Therefore, novel strategies are utmost importance for the patients due to the modest effect platinum-based therapy and the targeted therapies development.

In clinical, combination regiment like TKIs and chemotherapy or ICIs improved the progression-free survival or overall survival compared with monotherapy^4^^,^^7^. Whether in clinical or preclinical trials, the new combination strategies are emerging. We previously studied the combination of various compounds including nature product combined with chemotherapy drugs and chemotherapy drugs plus target drugs against different cancer cells^9–11^. In this research, a new combination of 10-Hydroxycamptothecin (HCPT) and crizotinib (CRI) was used to treat NSCLC. HCPT is a derivative of camptothecin, which has strong antitumor effect due to the selectively inhibitory of DNA topoisomerase I leading to disturb the duplication of DNA. The synergistic effects on lung cancer cells were observed after treated with combination of HCPT and other agents such as Paris Saponin I^10^, Astragalus polysaccharide^12^, and triptolide^13^. However, it is unclear whether the combination of HCPT and targeted drugs has synergistic anticancer effect. CRI is a TKI of ALK, c-Met, and ROS1^14^, and is the standard treatment for advanced NSCLC with ALK rearrangements. It was reported that CRI alone or combination with EGFR-TKI produced effective therapeutic for EGFR-mutant NSCLC patients^15^. CRI combined with gemcitabine and carboplatin could effectively improve quality of life, prolong the long-term survival rate, and present a good effect on advanced NSCLC patients without gene mutation compared with single chemotherapy^16^. We for the first time explored the effect and mechanism of HCPT combined with CRI on EGFR mutated adenocarcinoma cells and large cell lung cancer cells. Based on the synergy of HCPT and CRI, we synthesised their conjugates and evaluated their anti-lung cancer activity.

## 2. Materials and methods

### 2.1. Cell culture and chemicals

Human non-small cell lung carcinoma cell line NCI-H460 (H460) were kindly presented by Prof. Zhi Yao, Human lung adenocarcinoma cells NCI-H1975 (H1975) and NCI-HCC827 (HCC827) were available from Fox Biotechnology Co., Ltd. Human umbilical vein endothelial cell HUVEC were obtained from Shanghai institute of biological science, Chinese academy of science (Shanghai, China). Human bronchial epithelial cell BEAS-2B were provided by Prof. Shuli Man (Tianjin University of Science and Technology, Tianjin, China). All lung cancer cells were cultured in Roswell Park Memorial Institute (RPMI) 1640 medium (Thermo-Fisher Scientific, Waltham, MA), supplemented with 10% foetal bovine serum (FBS), 100 U/mL penicillin and 100 μg/mL streptomycin at 37 °C in a 5% CO_2_ humidified incubator. HUVEC cells were grown in DME/F-12 and BEAS-2B cells were cultured in High Glouse medium with 1% penicillin-streptomycin solution and 10% foetal bovine serum were added.

10-hydroxycamptothecine (HCPT, > 98%) and Crizotinib (CRI, > 98%) were purchased from Myrell Chemical Technology Co. Ltd and Adama reagent Co. Ltd respectively. Both of them were dissolved in Dimethyl Sulphoxide (DMSO, Amresco-LIFE SCIENCE). The primary antibodies used in the experiment were as follows: Bax (5023), Bcl-2 (2870), Bcl-xL (2764), Cytochrome-C (Cyto-C, 11940), EGFR (D38B1), phospho-EGFR (p-EGFR, D83B1), AKT (4691), phospho-AKT (p-AKT, 4060), ERK (4695), phospho-ERK (p-ERK, 4370), JNK (9252), phospho-JNK (p-JNK, 4668), p38 MAPK (8690), phospho-p38 MAPK (p-p38 MAPK, 4511). All the above antibodies were purchased from Cell signalling Technology (CST) and α-Tubulin (T9026) was obtained from Sigma-Aldrich.

### 2.2. Cell viability assay

The inhibition of cell proliferation was measured by MTT assay. H460, H1975 and HCC827 cells were plated in 96-well plates at a density of 5 × 10^4^ cells/mL for 24 h at 37 °C. Cells were treated with different concentrations of HCPT, CRI or the combination for 48 h (H460) or 72 h (H1975 and HCC827). MTT (5 mg/mL) was added to each well for 4 h. DMSO was used to dissolve the purple formazan crystals after removing MTT solution. Then the absorbance was measured at 492 nm and 630 nm. The half of the inhibiting concentration (IC50) was calculated by GraphPad software. The toxicity of HCPT, CRI and the combination to BEAS-2B and HUVEC cells was also used MTT assay. The cell viability of compound **CH-1** and compound **CH-2** was measured by the same experimental steps. DMSO was used as the control group.

Combination index (CI) were obtained using CompuSyn software (ComboSyn, Inc.), and CI <1, CI = 1, CI > 1, represents a synergistic effect, additive effect, and antagonism, respectively.

### 2.3. DAPI staining

H460, H1975 and HCC827 cells were seeded into a six-well plate at a density of 5 × 10^4^ cells/mL for 24 h. The cells were treated with DMSO, HCPT (0.125 or 0.5 μM), CRI (1, 1.25, or 2 μM) or the combination for 48 h. Each well was washed by 1 × PBS and fixed with pre-cooled methanol for 30 min. The cells were stained with DAPI (1 μg/mL) for 30 min after removing methanol and then they were photographed under a fluorescence microscope (Nikon, Tokyo, Japan).

### 2.4. Cell colony formation assay

H460, H1975 and HCC827 cells (800 cells/well) were seeded into cell culture dish (60 mm) for 7 days. Then the cells were exposed to DMSO, HCPT (0.125 or 0.5 μM), CRI (1, 1.25, or 2 μM) or the combination for 5 days. Cells were stained with 0.1% crystal violet for 2 min and photographed. Image Pro Plus 6.0 software was used to count the number of clones, which more than 50 cells were generally regarded as one clone.

### 2.5. Apoptosis assay

H460, H1975 and HCC827 cells (5 × 10^4^ cells/mL) were plated into 6-well plate for 24 h. H460 cells was treated with DMSO, HCPT (0.125 or 0.5 μM), CRI (1, 1.25, or 2 μM) or the combination for 24 h and 48 h, while H1975 and HCC827 cells were treated for 48 h and 72 h. Then cells were stained with Annexin V (5 μL) for 10 min and propidium iodide (PI, 5 μL) for 15 min in the dark. Immediately, the cells were analysed by a FACS Calibur flow cytometer (BD Biosciences, San Jose, CA).

### 2.6. Detection of JC-1 mitochondrial membrane potential

H460, H1975 and HCC827 cells were added to 6-well plates overnight and were exposed to DMSO, HCPT (0.125 or 0.5 μM), CRI (1, 1.25, or 2 μM) or the combination for 48 h (H460 cells) or 72 h (H1975 and HCC827 cells). Then cells were washed by 1 × PBS and fixed with pre-cooled methanol for 30 min followed by stained with the working solution of JC-1 for 15 min. After removing the working solution of JC-1, the cells were photographed under a fluorescence microscope (Nikon, Tokyo, Japan).

### 2.7. Western blot

H460, H1975 and HCC827 cells were exposed to DMSO, HCPT (0.125 or 0.5 μM), CRI (1 or 2 μM) or the combination for 48 h or 72 h. The proteins were collected after cell lysis and centrifugation. The protein concentration was determined by Coomassie blue method. The proteins were separated by 10% SDS-PAGE and transferred to PVDF membrane. After blocked with 5% skimmed milk, the membranes were immunoblotted with the primary antibodies (dilution of 1:1000) against Bax, Bcl-2, Bcl-xL, Cyto-C, Casp-8, Cleaved-Casp-8, EGFR, p-EGFR, AKT, p-AKT, ERK, p-ERK, JNK, p-JNK, p38 MAPK, p-p38 MAPK and α-Tubulin overnight at 4 °C. Then secondary antibodies-conjugated with horseradish peroxidase (HRP) were applied (dilution of 1:2000) in blocking buffer for 1 h. HRP -conjugated goat anti-rabbit or anti-mouse IgG was used as a secondary antibody for enhanced chemiluminescence (Invitrogen). The blots were visualised by Odyssey infra-red imaging system (LI-COR Biotechnology, USA).

### 2.8. General procedure for the synthesis of coupling compounds

#### 2.8.1. Compound CH-1

(R)-4–(4-(4–(6-amino-5–(1-(2,6-dichloro-3-fluorophenyl)ethoxy)pyridin-3-yl)-1H-pyrazol-1-yl)piperidin-1-yl)-4-oxobutanoic acid (compound **1**) was obtained by the reaction of CRI with succinic anhydride. HCPT (100 mg, 0.27 mmol) and compound **1** (226.60 mg, 0.41 mmol) were dissolved in 2 mL of DMF. Mixture was further charged with DMAP (2.01 mg, 16.47 μmol), DIEA (0.11 mL, 0.69 mmol) and EDCI (105.23 mg, 0.55 mmol, dissolved in DCM) at 0 °C and continuously stirred overnight at 30 °C. The mixture was extracted with dichloromethane (3 × 100 mL), washed with saturated salt water and dried over anhydrous sodium sulphate to obtain the crude product that was further purified by silica gel column chromatography using gradient dichloromethane: methanol (100:1–25:1) as the eluent. Characterisation of compound **CH-1** is given below.

Faint yellow solid, 201 mg, yield 82%. ^1^H NMR (400 MHz, CDCl_3_) *δ* 8.27 (s, 1H), 8.19 (d, *J* = 9.2 Hz, 1H), 7.67 (s, 2H), 7.65 (s, 1H), 7.59 (dd, *J* = 9.2, 2.4 Hz, 1H), 7.53 (s, 1H), 7.45 (s, 1H), 7.29 − 7.26 (m, 1H), 7.03 (t, *J* = 8.4 Hz, 1H), 6.83 (s, 1H), 6.03 − 6.02 (m, 1H), 5.72 (d, *J* = 16.4 Hz, 1H), 5.29 (d, *J* = 16.4 Hz, 1H), 5.25 (s, 2H), 5.03 (s, 2H), 4.74 (d, *J* = 14.0 Hz, 1H), 4.36 − 4.31 (m, 1H), 4.06 (d, *J* = 13.6 Hz, 1H), 3.74 (t, *J* = 6.4 Hz, 1H), 3.27 (t, *J* = 12.0 Hz, 1H), 2.99 (t, *J* = 6.4 Hz, 2H), 2.88 − 2.82 (m, 3H), 2.26 − 2.16 (m, 2H), 2.04 − 1.94 (m, 2H), 1.92 − 1.88 (m, 2H), 1.83 (d, *J* = 6.8 Hz, 3H), 1.03 (t, *J* = 7.6 Hz, 3H). ^13^C NMR (100 MHz, CDCl_3_) *δ* 174.0, 171.8, 169.5, 157.7, 152.5, 150.4, 149.9, 149.1, 147.0, 146.3, 140.1, 140.0, 137.0, 136.1, 134.8, 131.3, 130.7, 129.2, 129.1, 129.1, 128.6, 126.2, 122.9, 122.2 (d, *J* = 19 Hz), 120.2, 119.0, 118.9, 118.8, 117.0, 116.8, 115.2, 98.2, 72.9, 68.1, 66.4, 59.0, 50.2, 44.4, 41.1, 32.9, 31.8, 29.8, 28.2, 25.7, 19.0, 8.0. HRMS (+ESI-TOF) [M + H]^+^ *m/z* calcd for C_45_H_41_N_7_O_8_FCl_2_: 896.2372; found, 896.2356.

#### 2.8.2. Compound CH-2

(S)-4-ethyl-4-hydroxy-3,14-dioxo-3,4,12,14-tetrahydro-1H-pyrano[3′,4′:6,7]indolizino[1,2-b]quinolin-9-yl (4-nitrophenyl) carbonate (Compound **2**) was synthesised by the reaction of HCPT with phenyl p-nitrochloroformate. Compound **2** (150 mg, 0.28 mmol) and CRI (102.07 mg, 0.23 mmol) were dissolved in DMF (1.5 ml) and DCM (1.5 mL), and continuously stirred overnight at 30 °C. The mixture was extracted with dichloromethane (3 × 100 mL), washed with saturated salt water and dried over anhydrous sodium sulphate to obtain the crude product that was further purified by silica gel column chromatography using gradient dichloromethane: methanol (50:1–30:1) as the eluent. Characterisation of compound **CH-2** is given below.

Faint yellow solid, 173 mg, yield 73%. ^1^H NMR (400 MHz, CDCl_3_) *δ*: 8.32 (s, 1H), 8.23 (d, *J* = 9.2 Hz, 1H), 7.76 (d, *J* = 1.2 Hz, 1H), 7.71 (d, *J* = 2.4 Hz, 1H), 7.67 (s, 1H), 7.62 (dd, *J* = 9.2, 2.8 Hz, 1H), 7.59 (s, 1H), 7.53 (s, 1H), 7.31 (dd, *J* = 8.8, 4.8 Hz, 1H), 7.06 (t, *J* = 8.4 Hz, 1H), 6.88 (d, *J* = 1.2 Hz, 1H), 6.08 (q, *J* = 6.8 Hz, 1H), 5.74 (d, *J* = 16.4 Hz, 1H), 5.32 − 5.28 (m, 1H), 5.29 (s, 2H), 4.85 (s, 2H), 4.53 − 4.34 (m, 3H), 3.29 (t, *J* = 12.8 Hz, 1H), 3.13 (t, *J* = 12.8 Hz, 1H), 2.31 − 2.26 (m, 2H), 2.17 − 2.14 (m, 2H), 1.93 − 1.87 (m, 2H), 1.86 (d, *J* = 6.8 Hz, 3H), 1.04 (t, *J* = 7.6 Hz, 3H). ^13^C NMR (100 MHz, CDCl_3_) *δ*: 174.0, 157.7, 153.2, 152.4, 150.4, 150.4, 149.2, 146.8, 146.2, 140.0, 137.0, 136.2, 135.5, 131.2, 130.7, 129.2, 129.1, 129.0, 128.6, 126.3, 123.0, 122.1 (d, *J* = 19 Hz), 120.3, 118.9, 118.9, 118.7, 117.0, 116.7, 115.0, 98.2, 72.9, 72.90, 72.6, 66.4, 58.8, 50.1, 49.6, 43.8, 43.4, 32.5, 32.1, 31.8, 27.1, 19.0, 8.0. HRMS (+ESI-TOF) [M + H]^+^ *m/z* calcd for C_42_H_37_N_7_O_7_FCl_2_: 840.2110; found, 840.2128.

### 2.9. Statistical analysis

All data were presented as mean ± *SD*. Data analysis was performed by GraphPad Prism 7 software (GraphPad, San Diego). The statistical analysis was used Student’s *t*-test or Dunnett’s analysis by ANOVA. The significance of difference was indicated as *p < 0.05.

## 3. Results

### 3.1. The synergistic effect of HCPT and CRI on lung cancer cells

**Figure 1.**
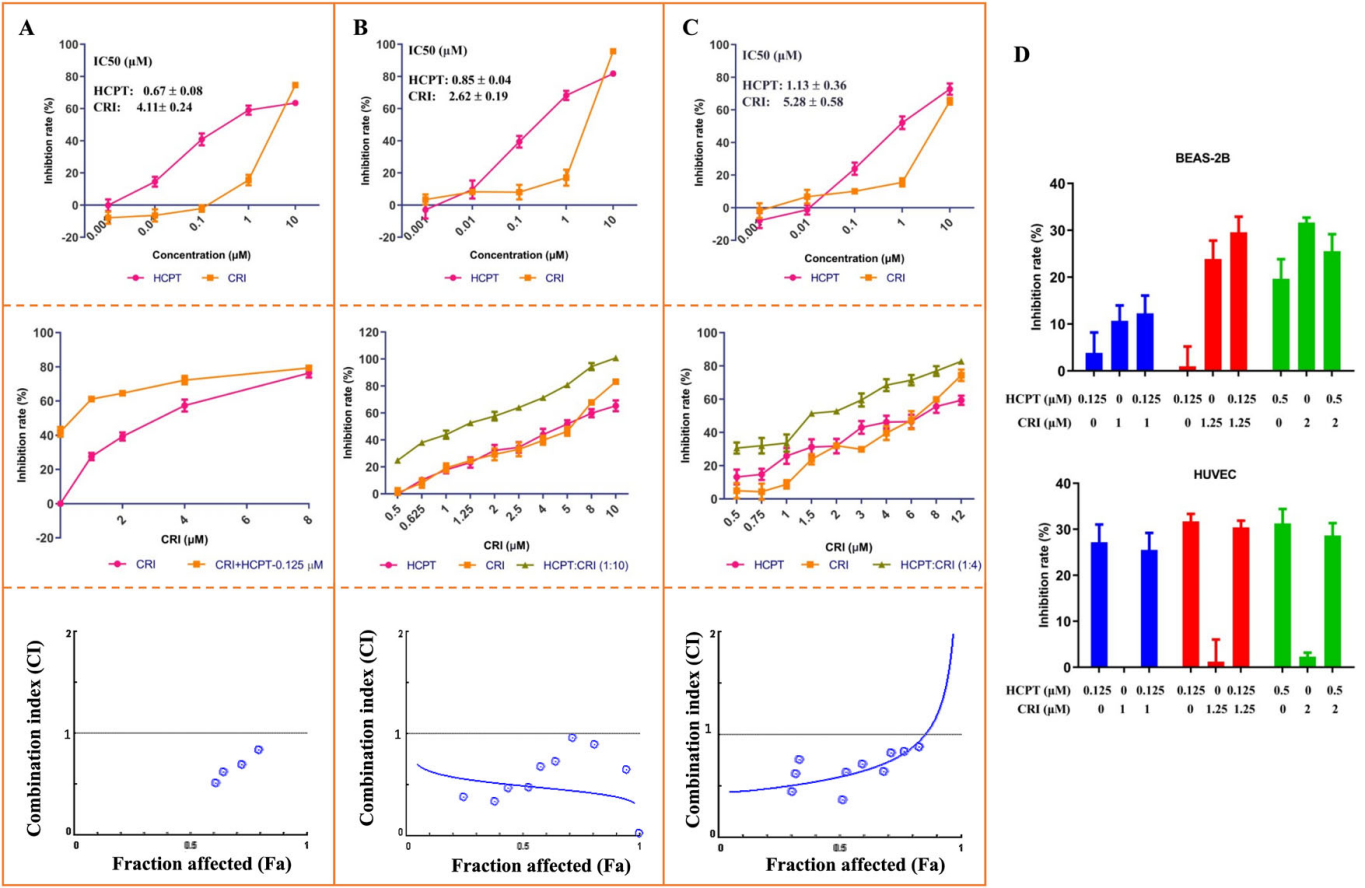
The cell viability of HCPT and CRI alone or in combination in H460 (A), H1975 (B), and HCC827 (C) cells as well as the toxicity to BEAS-2B and HUVEC cells (D). Cell viability was assessed by MTT assay. Cancer cells were treated with various concentrations (0, 0.001, 0.01, 0.1, 1, and 10 μM) of HCPT and CRI alone for 48 h (H460 cells) or 72 h (H1975 and HCC827 cells) (upper panel). For the middle panel, the lung cancer cells were treated with the combination of HCPT and CRI. CI values for the combination were calculated by the CompuSyn software (lower panel). HCPT (0.125 μM) combined with CRI (1, 2, 4, 8 μM) showed a synergistic effect on H460 cells. The combination of HCPT and CRI (0.5–12 μM) exhibited a synergistic effect on H1975 and HCC827 cells at the ratio of 1:10 and 1:4. IC_50_ was calculated by GraphPad software.

As shown in Figure 1, HCPT and CRI treatment significantly decreased the viability of lung cancer cells in a dose-dependent manner. HCPT or CRI showed significant cytotoxicity in H460 (Figure 1(A, upper panel)), H1975 (Figure 1(B, upper panel)) and HCC827 (Figure 1(C, upper panel)) cells with IC_50_ of 0.67 ± 0.08 μM, 4.11 ± 0.24 μM; 0.85 ± 0.04 μM, 2.62 ± 0.19 μM; and 1.13 ± 0.36 μM, 5.23 ± 0.58 μM, respectively. The combination of HCPT and CRI showed stronger inhibitory effect on the lung cancer cell lines than that of single drug (Figure 1(A–C, middle panel)) and CI values were less than 1 (Figure 1(A–C, lower panel)), which indicated that the combination of the two drugs synergistically inhibited the cell viability of H460, H1975, and HCC827 cells. Base on the results, we chose the concentration 0.125 μM HCPT and 1 μM CRI for H460 cells, 0.125 μM HCPT and 1.25 μM CRI for H1975 cells, 0.5 μM and 2 μM for HCC827 cells in the following experiments. Therefore, we first investigated the toxicity of HCPT and CRI alone or in combination at the above concentration on BEAS-2B and HUVEC cells. It was shown that the inhibition rate of all groups on BEAS-2B and HUVEC cells was less than 30% (Figure 1(D)), which mean the drugs had minor effect on the normal cell. The results indicated that HCPT and CRI alone or in combination could selectively affect the lung cancer cells.

**Figure 2.**
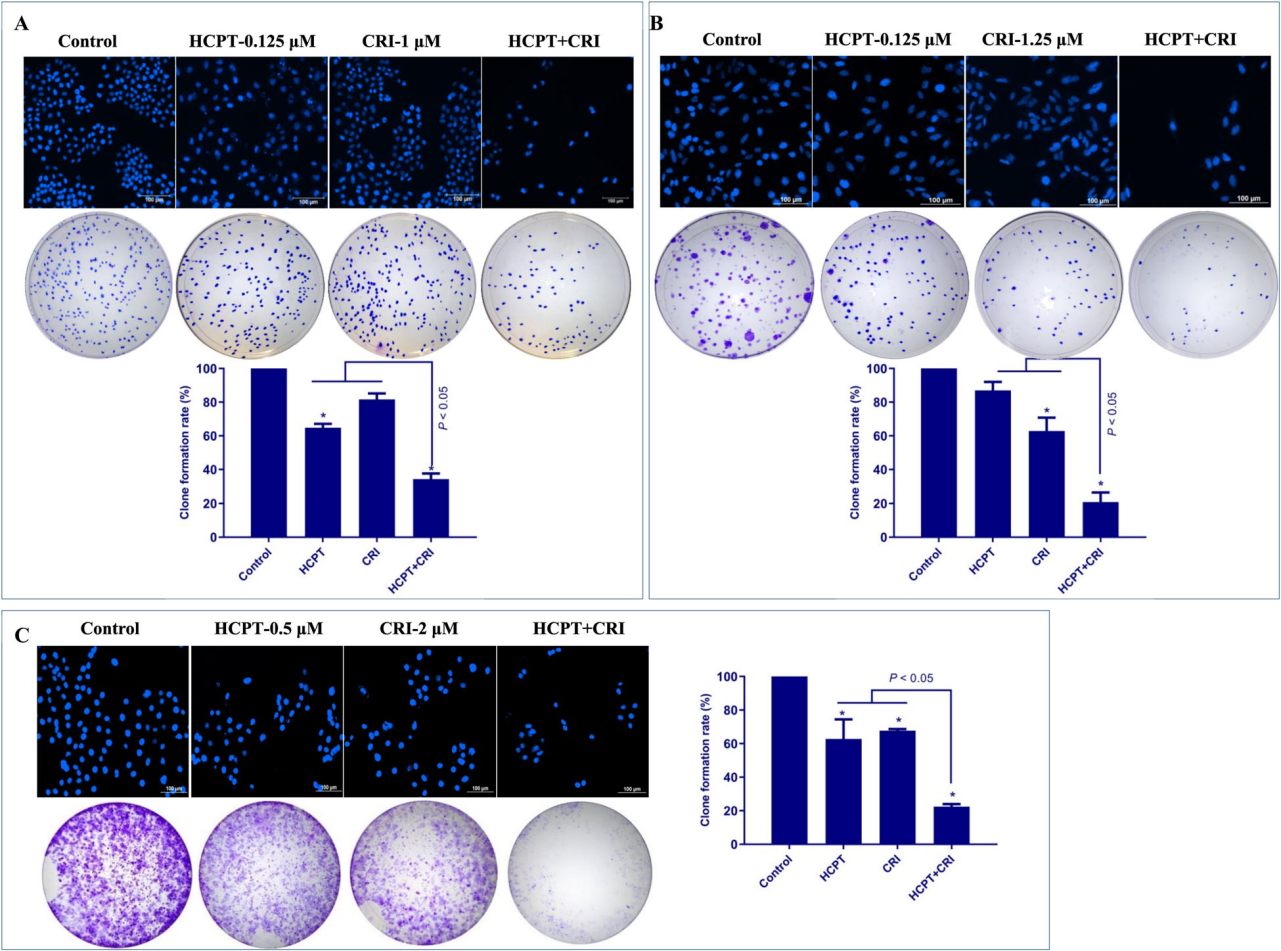
The morphological features of cancer cells and effect on cancer cells proliferation after HCPT and CRI alone or in combination treatment. The fluorescence intensity and colony formation of H460 (A), H1975 (B), and HCC827 (C) cells were significantly decreased when treated with HCPT and CRI. *p < 0.05, *vs* control group.

We further observed the cell morphology by DAPI staining and detected the cell proliferation by colony formation assay. As shown in Figure 2, the fluorescence intensity obviously decreased in combination group compared with single drug treatment groups, which reflected the number of lung cancer cells significantly reduced. The plate colony formation assays also showed similar results. HCPT combined with CRI remarkable inhibited the formation of cancer cell colonies, which was statistically significant compared with HCPT or CRI alone (*p* < 0.05). The above results suggested that the combination of HCPT and CRI could synergistically inhibit the growth and proliferation of lung cancer cells.

### 3.2. The combination of HCPT and CRI induced cell apoptosis through activation of mitochondria pathway

We used Annexin V-FITC/PI apoptosis detection kit to determine the number of apoptotic cells induced by HCPT and CRI alone or in combination. As shown in Figure 3 and Supplementary file Figure S1, the combination of HCPT and CRI induced stronger cell apoptosis than those agents alone. For H460 cells, the apoptosis rate after 48 h of combination treatment was 39.3%, while it was below 24% when treated with HCPT and CRI alone (Figure 3(A), *p* < 0.05). H1975 cells were sensitive to the combination especially for 72 h, and the apoptosis rate was 53.5% (Figure 3(B), *p* < 0.05). Similarly, HCPT combined with CRI significantly increased the cell apoptosis rate at 72 h (Figure 3(C), *p* < 0.05). Our results showed that the combination of HCPT and CRI exhibited the synergistic effect on the lung cancer cell by inducing cell apoptosis.

Figure 3.The effect of HCPT and CRI on cell apoptosis of H460 (A), H1975 (B), and HCC827 (C) cells. The percentage of apoptotic cells was estimated by Annexin V-FITC/PI kit. The mitochondrial membrane potential was detected by JC-1 method. Western blot was used to determine the expression of apoptosis related proteins. *p < 0.05, *vs* control group.

**Figure:**
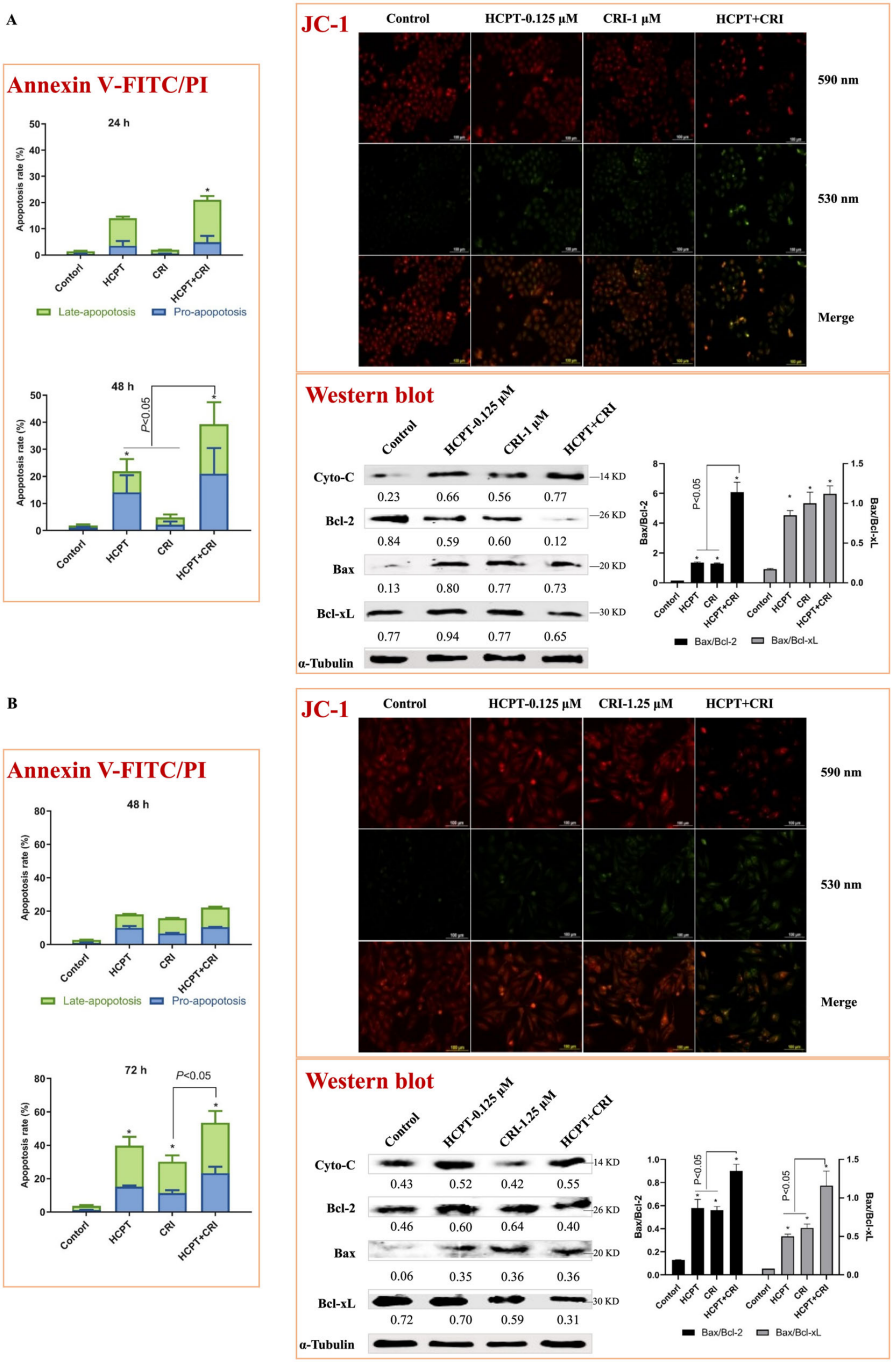

**Figure:**
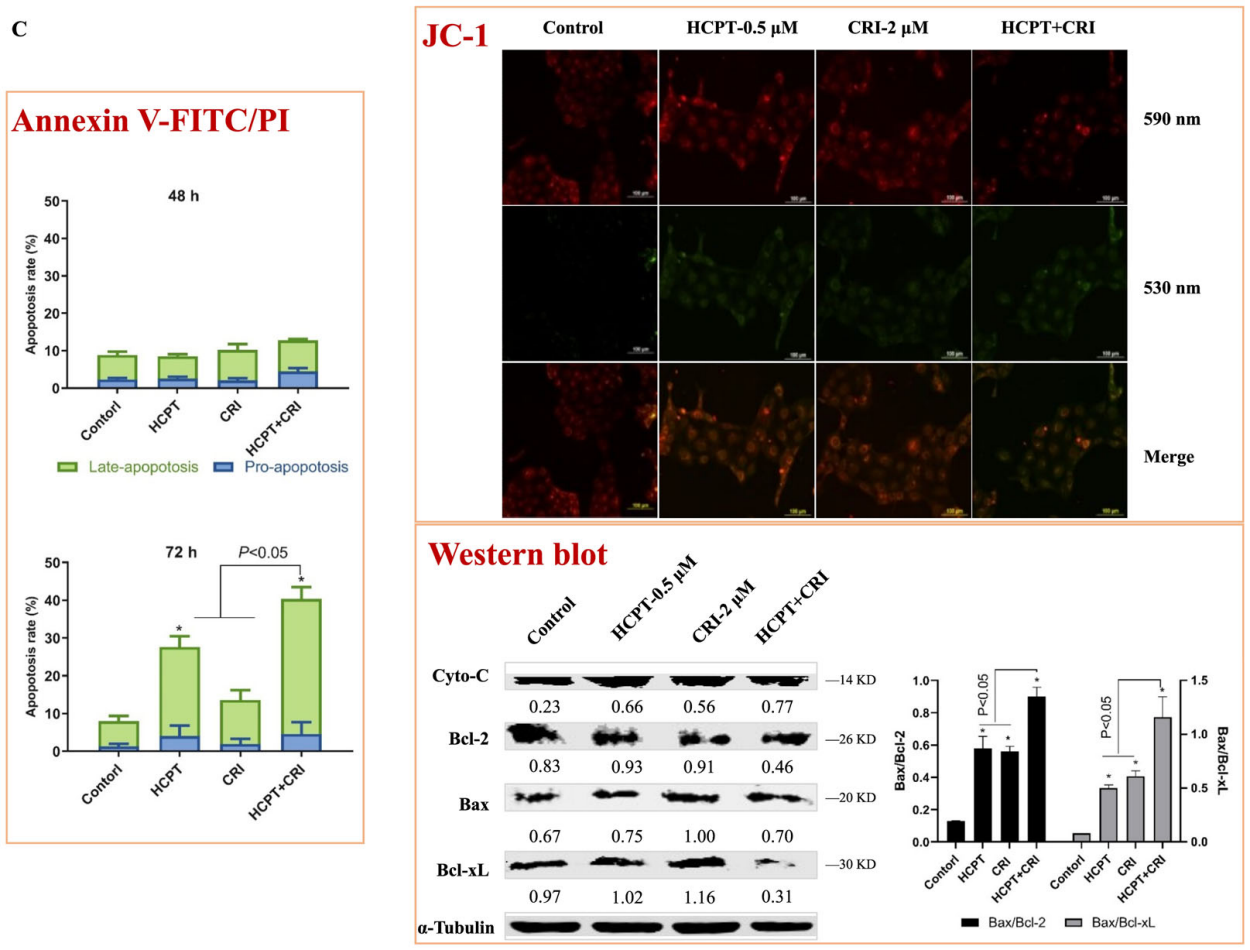

**Figure 4.**
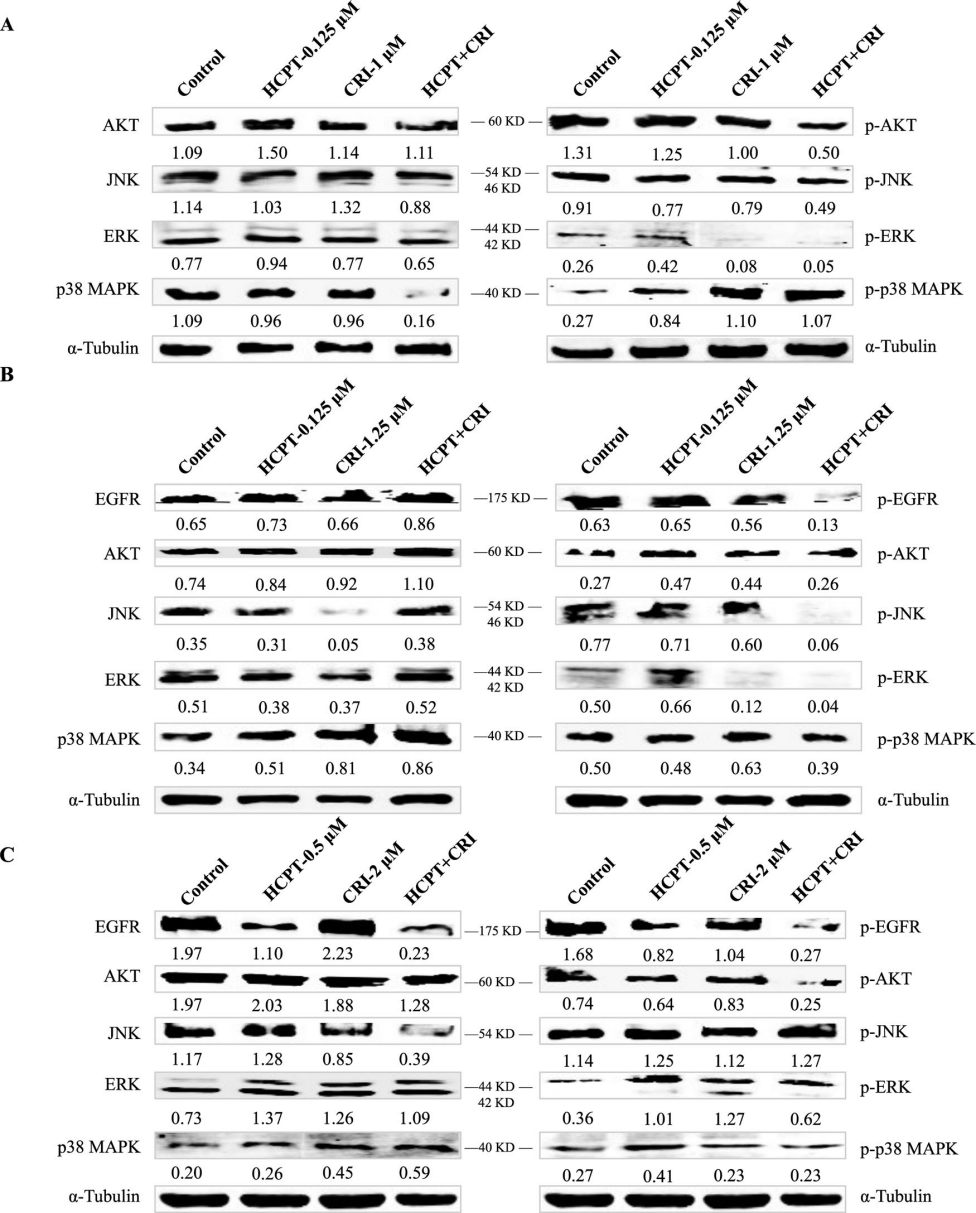
The regulation of HCPT and CRI on EGFR downstream signalling pathways in H460 (A), H1975 (B), and HCC827 (C) cells. The total proteins and phosphorylated proteins related to EGFR signalling pathway including AKT, JNK, ERK, and p38 MAPK were detected after treated with HCPT and CRI alone or in combination.

**Figure 5.**
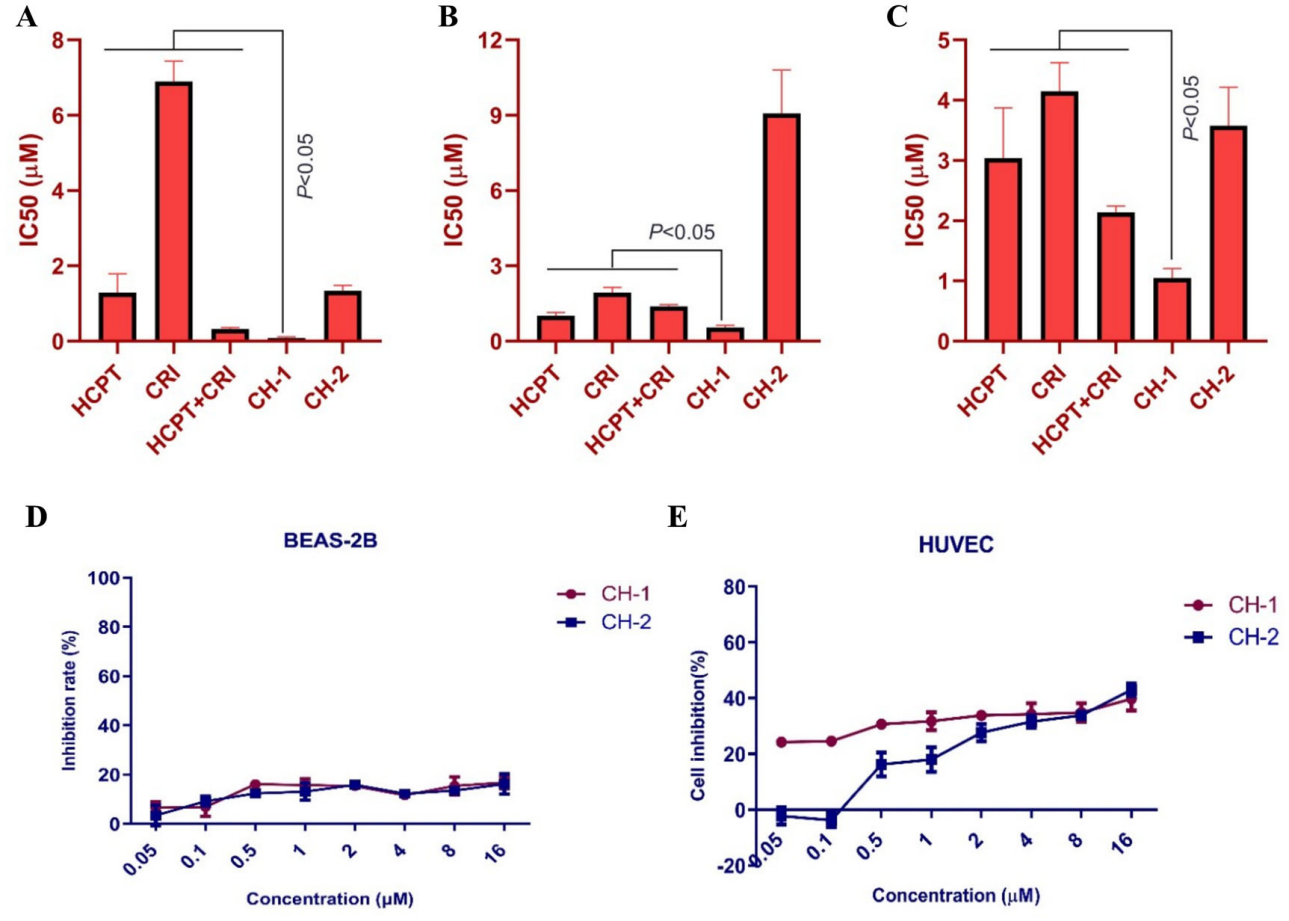
The *in vitro* effect of compounds **CH-1** and **CH-2** on lung cancer cells and normal cells. The cytotoxicity of compounds **CH-1** and **CH-2** on H460 (A), H1975 (B), and HCC827 (C) cells was dose-dependent. The compounds **CH-1** and **CH-2** had little toxicity to BEAS-2B and HUVEC cells (D and E).

Endogenous apoptosis is mainly realised through mitochondrial pathway, which is one of the most clearly clarified signal pathways. To investigate whether the combination of the two agents induced cell apoptosis was associated with mitochondrial pathway, we detected the mitochondrial membrane potential by the Cationic Dye JC-1, which could detect the integrity of mitochondrial membrane according to the content of J-monomers (typical green fluorescence, 530 nm) and the formation J-aggregates (with a specific red fluorescence at 590 nm)^17^. The control group of H460, H1975 and HCC827 cells contained mitochondria labelled with the fluorescence typical for both J-aggregates and J-monomer, but there were more changes from J-aggregates to J-monomer after the combination of HCPT and CRI (Figure 3, JC-1), which indicated that HCPT combined with CRI damaged the mitochondrial function and then decreased the mitochondrial membrane potential.

To further explore the internal mechanism of cell apoptosis induced by HCPT and CRI, the expression of apoptosis-associated proteins including Cyto-C, Bcl-2, Bax and Bcl-xL was analysed by western blot method. We found that HCPT and CRI alone or in combination significantly increased the release of Cyto-C and the level of Bax in the three lung cancer cell lines when compared with control group. However, the single agents had little effect on the levels of Bcl-2 and Bcl-xL, but the combination of HCPT and CRI could significantly decrease their expressions in H460, H1975 and HCC827 cells. Thus, the associated Bax/Bcl-2 ratio or Bax/Bcl-xL ratio was obviously upregulated after the combination treatment (Figure 3, Western blot). Our results suggested that the combination of HCPT and CRI induced the lung cancer cell apoptosis through releasing Cyto-C by activation of Bcl-2 family-mediated mitochondrial signalling.

### 3.3. The combination of HCPT and CRI inhibited the EGFR downstream signalling pathway

To further evaluate the synergistic effect of HCPT and CRT on lung cancer cells, the proteins associated EGFR downstream signalling pathway including AKT, ERK, JNK, EGFR and p38 MAPK were analysed by western blot. We found that the HCPT combined with CRI could significantly decreased the expression of p-AKT, p-JNK, and p-ERK, but reduced the level of p38 MAPK as well as increased its phosphorylated protein p-p38 MAPK in H460 cells (Figure 4(A)). As H460 cells are EGFR wild-type cells, the results mean that the activation of phosphorylated proteins of AKT, ERK and JNK were existed in the cancer cells and suppressed by the combination agents, which might be responsible for the synergistic effect of HCPT and CRI on H460 cells. Both of H1975 and HCC827 cells are EGFR mutation cell lines, thus we detected the expression of EGFR and p-EGFR. HCPT combined with CRI could significantly reduce the level of p-EGFR and slightly decrease the expression of p38 MAPK for H1975 and HCC827 cells. The effect of the combination on regulating p-AKT, p-JNK, and p-ERK in the two lung cancer cells were different. The combined agents could decrease the level of p-JNK and p-ERK in H1975 cell and suppress the expression of p-AKT in HCC827 cells (Figure 4(B,C)). The results indicated that HCPT combined with CRI exerted synergistic effects on the three lung cancer cells through EGFR related pathway.

### 3.4. Synthesis of compounds CH-1 and CH-2

**Scheme 1.**
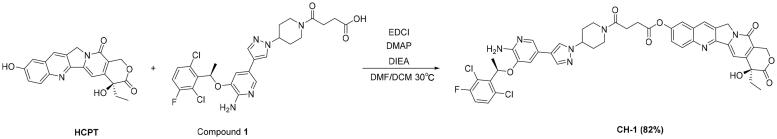
Synthesis of compound **CH-**1.

**Scheme 2.**
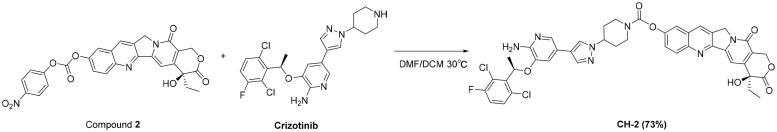
Synthesis of compound **CH-**2.

Our strategy was to obtain coupling compounds of HCPT and CRI through the combination of two separate pharmacophoric groups into one compound. The length of the connecting chain between compounds is related to the activity. Compound **CH-1** posed a long chain by connecting HCPT and CRI with succinic acid group and compound **CH-2** had a short chain by connecting them with carboxy group. The synthetic route started with the first reaction of the insertion of the –CO(CH_2_)_2_COOH group in CRI (compound **1**) and then reacted with HCPT in DMAP, DIEA and EDCI, obtaining compound **CH-1** in excellent yield (82%) (Scheme 1). We inserted the p-nitrophenyl formate group into HCPT and got compound **2**, which was then reacted with CRI under DMF and DCM solutions to obtain compound **CH-2** with a good yield (73%) (Scheme 2). The structure of the two compounds synthetised was confirmed by ^1^H and ^13^C nuclear magnetic resonance (NMR) spectroscopy and HRMS spectrometry experiments (Supplementary file Figure S2 and S3).

### 3.5. Compounds CH-1 and CH-2 inhibited cancer cell growth in vitro

Both of compounds **CH-1** and **CH-2** had a good inhibitory effect on the proliferation of the three lung cancer cells. When compared with HCPT and CRI alone or combination, compound **CH-1** exhibited significant inhibition on H460, H1975, and HCC827 cells with the IC_50_ of 0.08 μM, 0.55 μM, and 1.05 μM, respectively (*p* < 0.05) (Figure 5(A–C)). We also detected the toxicity of the two compounds for normal cells. Both of them had little effect on BEAS-2B and HUVEC cells with the inhibition rate less than 40% (Figure 5(D,E)).

## 4. Discussion

NSCLC is classified into adenocarcinoma, squamous cell carcinoma, and large cell cancer based on histology. With the discovery of driving gene mutations, a new classification paradigm of NSCLC characterised by EGFR, ALK and ROS1 has emerged^18^. Consequently, targeted therapies for oncogene-driver NSCLC have been continuously developed and approved especially for treatment of EGFR-mutation patients^4^. Mutations of EGFR are common drivers of oncogenesis in NSCLC, among which exon 19 deletions (ex19del) and exon 21 L858R point mutations (L858R) are the most common EGFR alterations^19^. EGFR-TKIs have been used for the treatment of advanced EGFR-mutated NSCLC. Three generations of EGFR TKIs demonstrated superiority clinical benefit compared with chemotherapy, including the first-generation TKIs, erlotinib and gefitinib; the second-generation TKIs, afatinib and dacomitinib; and the third-generation TKI osimertinib^3^^,^^4^. However, drug resistance is inevitable after using TKIs. The T790M mutation is the commonest mechanism of resistance to first- and second generation EGFR-TKIs^20^. Osimertinib was approved for the treatment of T790M positive NSCLC in a second line, subsequently in the front line for common EGFR mutations, and now it is a standard of care in many countries for above indications^3^. Unfortunately, EGFR secondary mutations like C797S, L718, and L792 confer osimertinib resistance and alterations in parallel or downstream oncogenes such as Met and KRAS contributed to the osimertinib-resistance in patients without EGFR secondary mutations^21^. Thus, various ongoing trials adopt the EGFR-TKI-based combination treatments to improve patient outcomes^4^^,^^7^.

We previously reported the mechanism of the combination of vinorelbine and afatinib (second-generation EGFR-TKI) against NSCLC^11^. In this study, we found that another combination agents HCPT and CRI showed synergistic activity on the same lung cancer cell lines including H460 (KRAS, large cell lung cancer), H1975 (L858R/T790M+, lung adenocarcinoma), and HCC827 (E746-A759del/T790M-, lung adenocarcinoma) cells. In terms of combination mode, chemotherapy combined with targeted therapy is a common strategy to improve patient outcomes in clinical trials, although conventional chemotherapy produced moderate clinical benefit. Adding pemetrexed and carboplatin chemotherapy to gefitinib significantly increased patient survival^22^. The combination of osimertinib and carboplatin-pemetrexed showed tolerable to patients with EGFR-mutated NSCLC^23^. In our study, HCPT combined with CRI could synergistically suppressed the short-term and long-term proliferation of NSCLC without affecting the normal cell growth at the appropriate doses (Figures 1 and 2). It was found that this synergistic effect was related to the combination induced mitochondrial cell apoptosis pathway (Figure 3). As mentioned, HCPT is an inhibitor of DNA topoisomerase I and CRI mediates the antitumor activities through targeting ALK, c-Met, and ROS^14^. From this point of view, it seems that the two drugs are not associated with EGFR- or KRAS-mutated NSCLC, but how to play the synergistic effect is worth exploring. Driver gene mutations are not only the targets of therapy, but also the biomarkers of predicting the efficacy of therapy. For example, drug resistance will lead to new mutations. A large number of clinical studies have shown that the mechanism of EGFR (or ALK)-TKI resistance are related to EGFR (or ALK) kinase domain mutations or activation of bypass signalling pathways^3^^,^^4^^,^^7^. For ALK, EGFR is its bypass signalling pathways and vice versa. Moreover, the mutations activate the similar intracellular signalling cascade including phosphatidylinositol 3 kinase (PI3K)/AKT, MEK/extracellular regulated protein kinases (ERK)/mitogen-activated protein kinases (MAPK), C-Jun terminal kinase (JNK), signal transducer and activator of transcription (STATs), nuclear factor-kappa B (NF-κB), and so on, resulting in DNA synthesis and cell proliferation^6^. Although the three cells used in our experiment contained different mutations, H1975 cells harbour the L858R/T790M mutation, HCC827 has an EGFR deletion mutation in exon 19, and H460 is KRAS mutation, the downstream signal pathways have changed to varying degrees. Therefore, we speculated that the combination of HCPT and CRI might play a synergistic anti-lung cancer effect by regulating the downstream pathways. The results showed that the HCPT combined with CRI suppressed the expressions of p-AKT, p-JNK, and p-ERK, and improved the level of p-p38 MAPK in H460 cells (Figure 4(A)). For common EGFR mutation cells HCC827, the combination of HCPT and CRI decreased the levels of p-EGFR, p-AKT, and p-p38 MAPK, and upregulated the level of p-ERK (Figure 4(C)). While, the two drugs downregulated the expressions of p-EGFR, p-JNK, and p-p38 MAPK, in T790M mutation cells H1975 (Figure 4(B)). The different proteins regulated by the combination also indirectly explain the different changes of these signal pathway proteins in these lung cancer cells. Our results indicated that the combination of HCPT and CRI inhibited the different lung cancer cells growth and induced the cell apoptosis through regulating the EGFR-related downstream signalling pathways, which was similar to the previously reported^11^. Some clinical cases reported that CRI was effective on the EGFR-mutant lung cancer patients^15^^,^^24^. Consistent with our research, CRI combined with other drugs exhibited the synergistic effect by inhibiting the activation of AKT or EKR in cancer cells^25^^,^^26^. It was found that JNK was a downstream signalling pathway of ALK^27^, and ROS1 signalling could activate apoptosis-related molecules p38 MAPK^28^. In addition, CRI is the inhibitor of ALK and ROS1, which explained why the combination of CRI and HCPT inhibited the expressions of JNK and p38 MAPK in this experiment. Subsequently, we applied the chemical synthesis to connect the two drugs into a conjugate with different chemical bonds. Combining different drugs through chemical links is a strategy for discovering new drugs, like antibody-drug conjugates (ADCs), which are immunoconjugates comprised of a monoclonal antibody tethered to a cytotoxic drug and nine ADCs are used in the marked^29^. When it comes to the simultaneous application of two or more chemotherapeutic agents, it is more inclined to study the transport of new delivery systems to tumour cells^30^. Recently, small molecule-drug conjugates have become an effective strategy for targeted delivery in cancer therapy, and the hybrids composed of some active components showed good activity^31–33^. We synthesised two compounds **CH-1** and **CH-2**, which had cytotoxicity *in vitro*. Compound **CH-1** obviously inhibited the lung cancer cell growth, which showed stronger cytotoxicity than the combination of the two drugs, but not **CH-2** (Figure 5). The difference between compounds is that the linker of **CH-2** is a short chain ester group, while it has one more -COCH2CH2- in **CH-1**, which might be the reason why the compound had anti lung cancer effect and no toxicity was observed. At present, we have preliminarily confirmed that the effect of the two drugs through chemical link was stronger than that of the combination. However, further study should be explored to verify the efficacy and toxicity of compound **CH-1**, as well as more appropriate linkers for HCPT and CRI.

Collectively, we for the first time investigated the effect of the combination of HCPT and CRI on EGFR- and KRAS-mutant lung cancer cells and first synthesised the conjugates of the two drugs. HCPT combined with CRI possessed the synergistic inhibition of proliferation and inducing cell apoptosis by inactivating of EGFR related downstream signalling pathways and the conjugate **CH-1** significantly suppressed the lung cancer cell growth with no observed toxicity. Our findings provided new insights supporting the utility of CRI for treating NSCLC with or without EGFR mutation and the combination of HCPT and CRI might be a promising therapeutic regimen for NSCLC. Meanwhile, the study successfully synthesised the conjugate **CH-1** offering a further basis for small molecule-drug conjugate as delivery system.

## Supplementary Material

Supplemental Material
