# Supplementary material for: Development of 10-Hydroxycamptothecin-crizotinib conjugate based on the synergistic effect on lung cancer cells
Source: J Enzyme Inhib Med Chem. 2022 Oct 28;38(1):1–11. doi: 10.1080/14756366.2022.2132487 (PMC9621280; doi:10.1080/14756366.2022.2132487)
Supplement: Supplemental Material [file IENZ_A_2132487_SM4824.pdf]

Development of 10-Hydroxycamptothecin-crizotinib conjugate based on the synergistic effect on lung cancer cells

Zhen Liu[a*1], Ye Xu[a1], Lvqian Guo[a], Xinran Li[a], Junling Gao[a], Weiran Xie[a], Lianbo Zhao[a], Yuou Teng[a], Xuejiao Li[b*], Peng Yu[a*]

a China International Science and Technology Cooperation Base of Food Nutrition/Safety and Medicinal Chemistry, Key Laboratory of Industrial Fermentation Microbiology of Ministry of Education, Tianjin Key Laboratory of Industry Microbiology, College of Biotechnology, Tianjin University of Science & Technology, Tianjin, 300457, P. R. China

b Central Laboratory, Endocrine and Metabolic Disease Center, The First Affiliated Hospital and College of Clinical Medicine of Henan University of Science and Technology; Medical Key Laboratory of Hereditary Rare Diseases of Henan; Luoyang sub-center of National Clinical Research Center for Metabolic Diseases, Luoyang, 471003, P. R. China

1 The two authors contributed equally to this work.

Corresponding author:

Dr. Zhen Liu, College of Biotechnology, Tianjin University of Science & Technology, Tianjin 300457, P. R. China, Telephone: 86-22-60912562; E-mail: liuzhen5957@163.com; liuzhen5957@tust.edu.cn

Dr. Xuejiao Li, The First Affiliated Hospital and College of Clinical Medicine of Henan University of Science and Technology, Luoyang, 471003, P. R. China, Telephone: 86-13502091956; E-mail: white_wnow1987@163.com

Prof. Peng Yu, College of Biotechnology, Tianjin University of Science & Technology, Tianjin 300457, P. R. China, Telephone: 86-22-60912562; E-mail: yupeng@tust.edu.cn

Information Classification: General

Supplementary file:

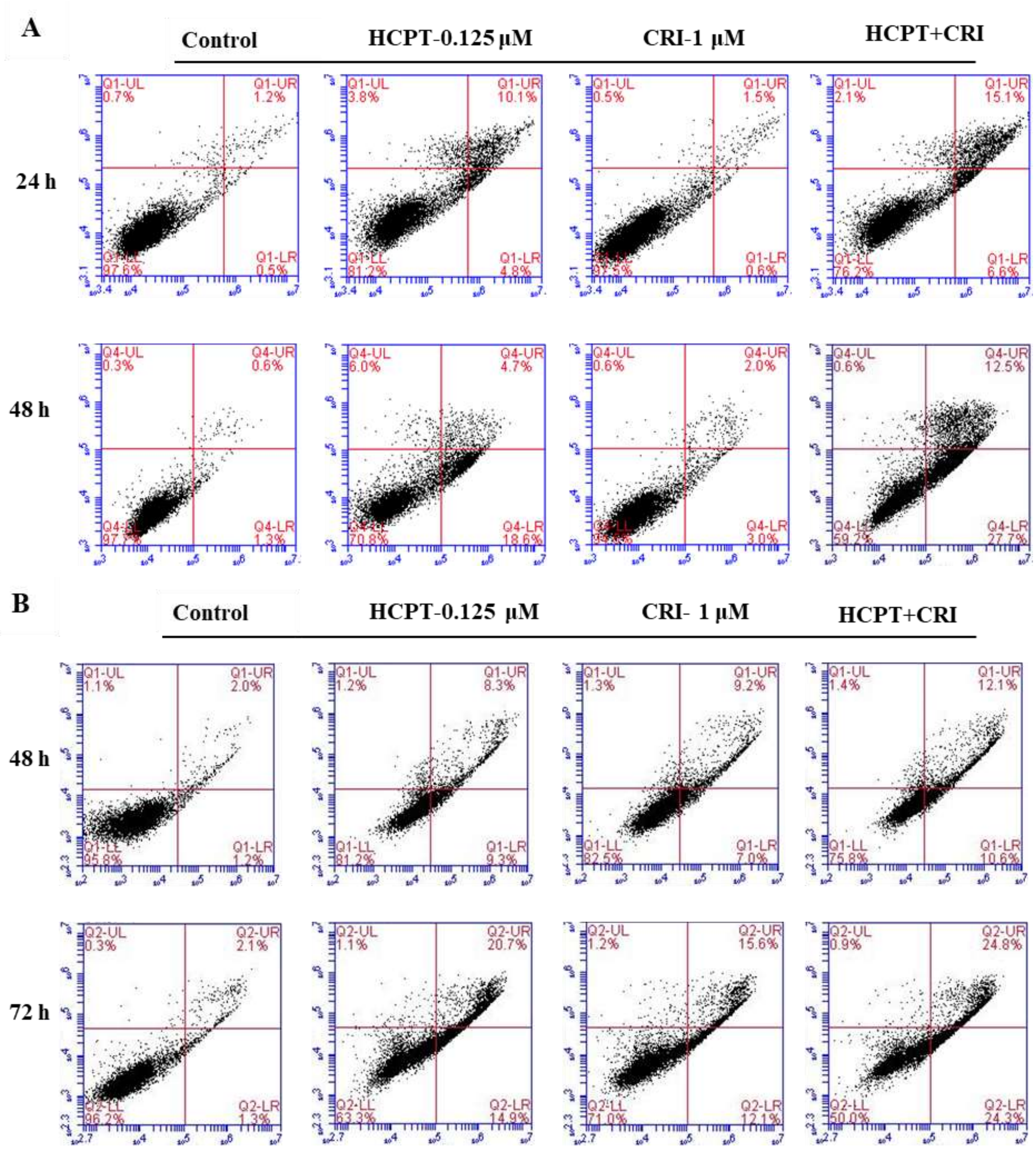

Information Classification: General

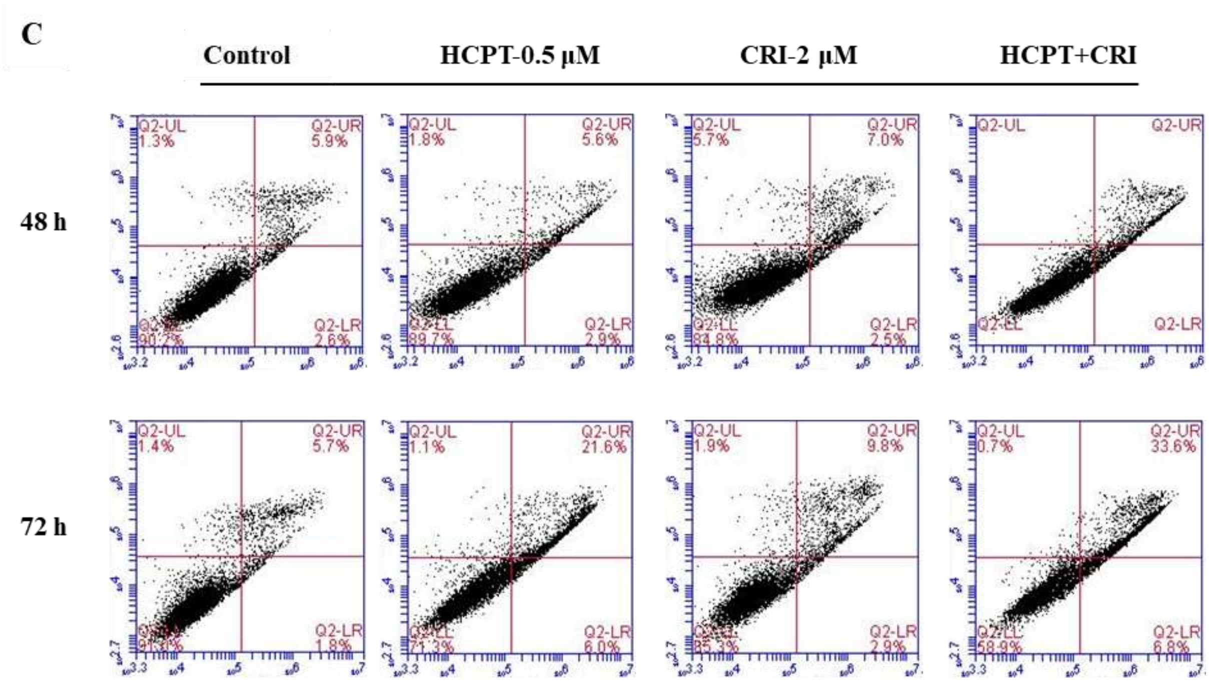

Figure S1. The effect of HCPT and CRI on cell apoptosis of H460 (A), H1975 (B), and HCC827 (C) cells. The apoptosis assay was estimated by Annexin V-FITC/PI kit and detected by flow cytometer.

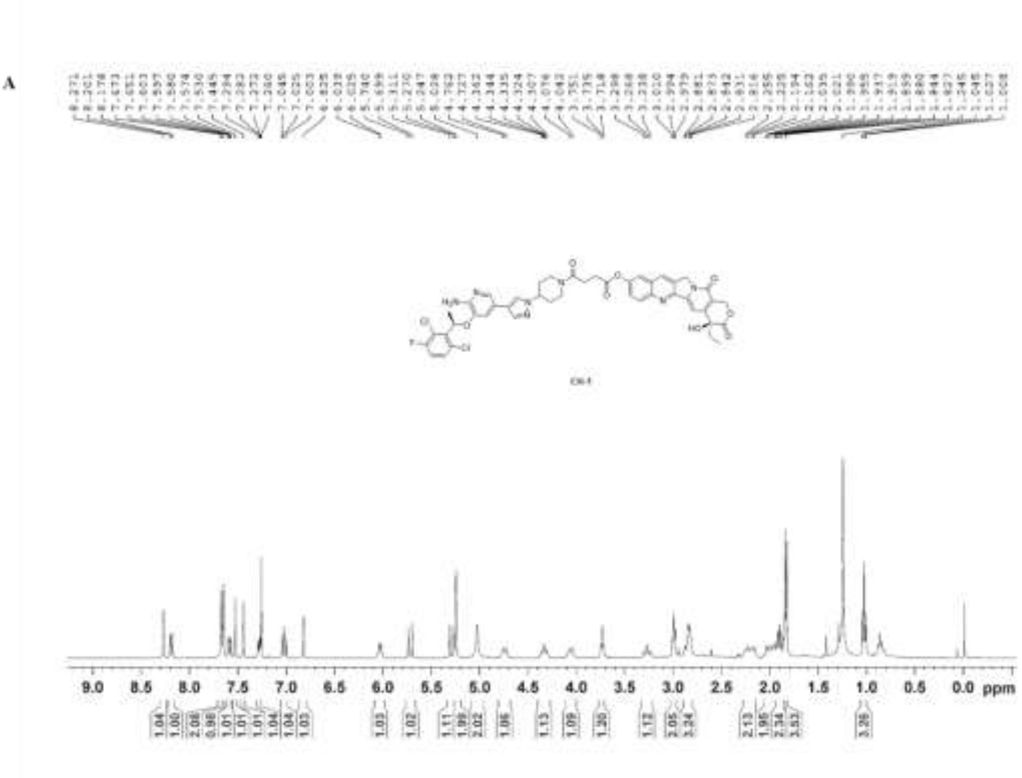

Information Classification: General

B

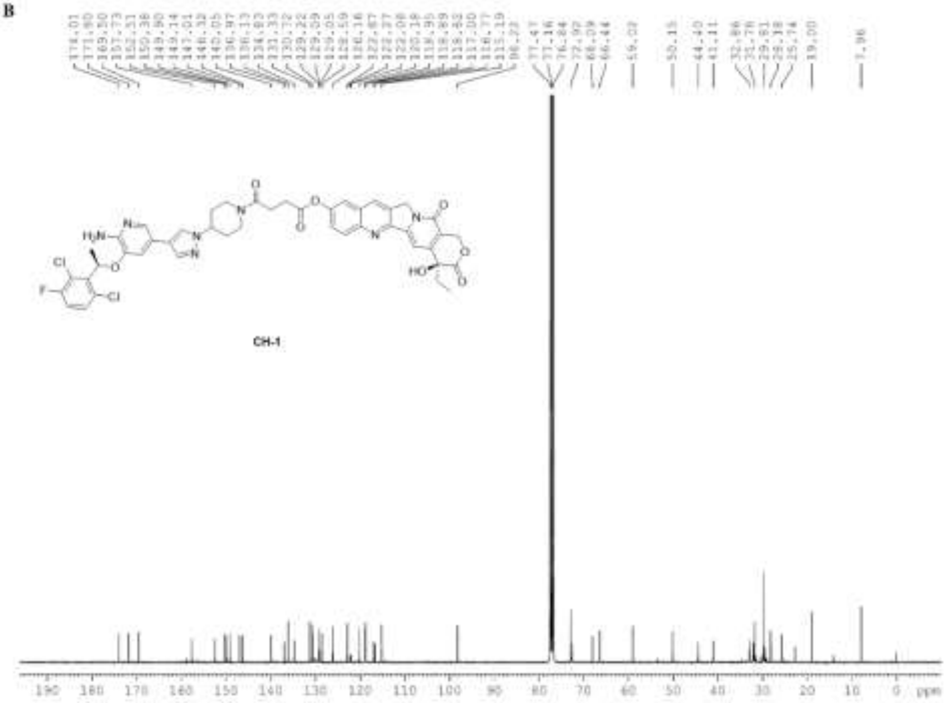

Figure S2. ^{1}H (A) and ^{13}C (B) NMR of compound **CH-1** in $CDCl_3$ solution

A

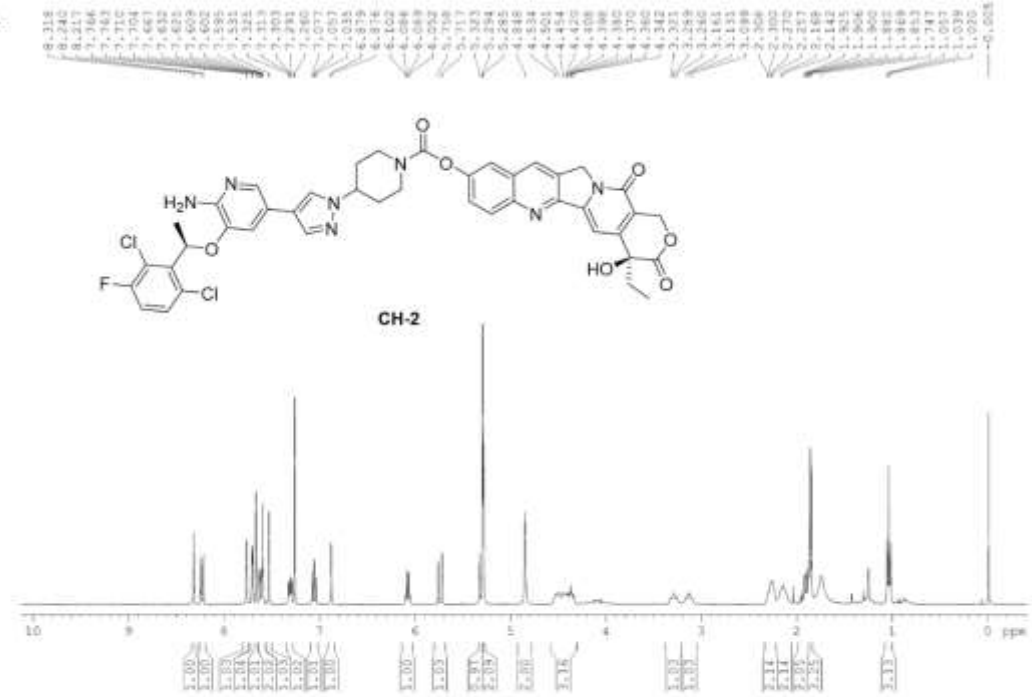

Information Classification: General

B

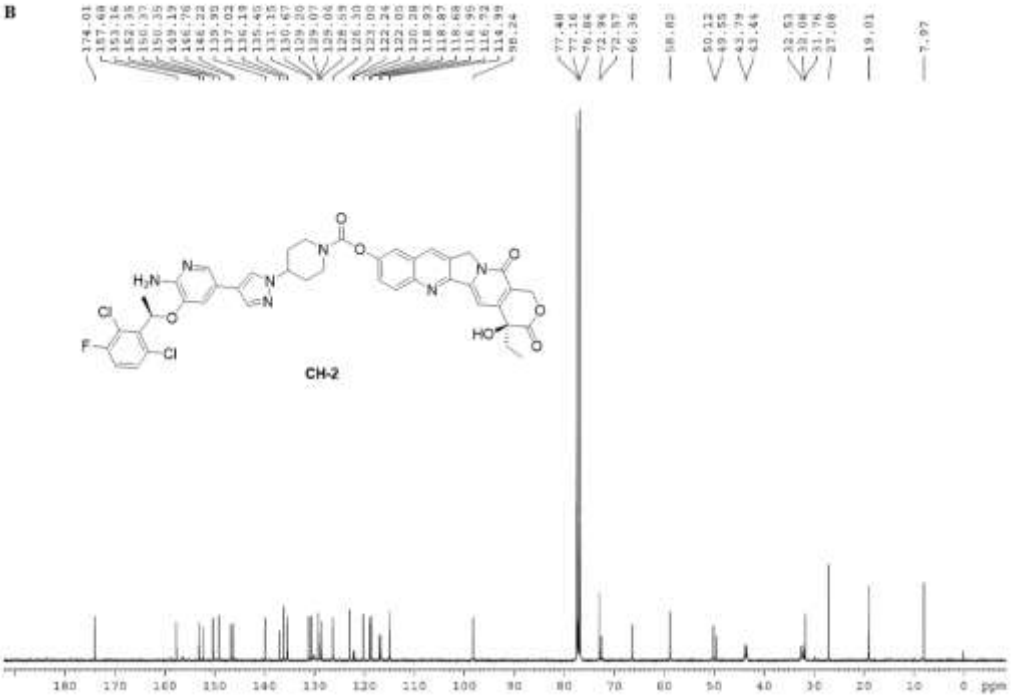

Figure S3. Fig. S2. ^{1}H (A) and ^{13}C (B) NMR of compound **CH-2** in $CDCl_3$ solution

Information Classification: General
